# Editorial: Microbial communities in fermented products: current knowledge and future prospects

**DOI:** 10.3389/fmicb.2024.1408480

**Published:** 2024-04-26

**Authors:** Photis Papademas, Spiros Paramithiotis, Maria Aspri, Thomas Bintsis, Dimitrios A. Anagnostopoulos, Dimitrios Tsaltas

**Affiliations:** ^1^Department of Agricultural Sciences, Biotechnology & Food Science, Faculty of Geotechnical Sciences and Environmental Management, Cyprus University of Technology, Limassol, Cyprus; ^2^Department of Biological Applications and Technology, School of Health Sciences, University of Ioannina, Ioannina, Greece; ^3^School of Veterinary Medicine, Faculty of Health Sciences, Aristotle University of Thessaloniki, Thessaloniki, Greece; ^4^Department of Ichthyology and Aquatic Environment, School of Agricultural Sciences, University of Thessaly, Volos, Greece

**Keywords:** fermentation, microbial communities, microbial interaction, microbial metabolites, culture-dependent techniques, culture-independent techniques, next generation sequencing

Fermentation has been applied since antiquity for the production of palatable food with excellent preservation capacity. It is driven by microbial consortia that are adapted to the nutrient and energy sources of the raw materials and specific ambient conditions. Through the interplay between abiotic and biotic factors, the production of fermented products with enhanced organoleptic characteristics, preservation potential and nutritional value, is achieved.

Assessment of the microbial communities that drive fermentation has attracted significant scientific attention. Characterization of the physicochemical properties of the raw materials employed, identification of the microorganisms that dominate or participate as secondary microbiota in these microecosystems, their metabolic potential, as well as the effect that the fermentation conditions may have on the biotransformations that take place during fermentation, have been in the epicenter of scientific scrutiny for decades. The aim of this Research Topic was to provide with a collection of articles that would facilitate our understanding on the aforementioned topics, identify research gaps and pave the road for future research.

The paramount importance of the raw materials and their effect on the physicochemical, sensorial, and functional quality of the final product was highlighted by the studies of Asif et al. and Wei et al.. In the first study, buttermilk of increasing fat content was used for the production of Cheddar-type cheese. Increase of the fat content resulted in the increase of the lactic, propionic, acetic, and citric acids, vitamins A and E, as well as the free fatty acid content, after 90 days of ripening. In addition, the highest fat content was awarded with the highest texture and sensory scores. Wei et al. studied the effect of broken egg addition to the composting mixture of *Pleurotus floridanus* cultivation, on the physicochemical properties and the bacterial microcommunities of the substrate as well as the agronomic and nutritional properties of the fruiting bodies. The physicochemical properties of the substrate after composing ranged within values that would not affect negatively the mycelial growth ratio and mushroom yield. After composting, the bacterial communities of the control and the compost made with the addition of broken eggs mixture consisted of members of the phyla Actinobacteriota, Firmicutes, and Proteobacteria. In all cases, the genera *Streptococcus* and *Streptomyces* seemed to prevail before composting and the genus *Acinetobacter* after composting. Regarding the agronomic properties of the fruiting bodies, addition of broken eggs mixture above 16.8 Kg, resulted in the increase of incubation period and contamination rate and the decrease of yield of first flush, total yield and biological efficiency; therefore, it was regarded as not suitable for *Pleurotus floridanus* cultivation. On the other hand, the addition of broken eggs resulted in the improvement of the nutritional quality and flavor of the fruiting bodies.

The characterization of the microbiota that is involved in fermentation, necessarily includes their identification at species or subspecies level and the assessment of their metabolic properties that are associated with the safety, the technological and the functional characteristics of the product. The genetic and technological diversity of dairy lactic acid bacteria isolates has been extensively assessed. Their probiotic potential has been in the epicenter of intensive study, since fermented dairy products are excellent vehicles for probiotic delivery. The latest research regarding the health benefits attributed to probiotic cultures, their mode of action, as well as the emerging applications in the food industry, were collected and comprehensively presented by Latif et al.. In addition, the probiotic potential of 23 lactic acid bacteria strains isolated from different fermented food products was presented by Megur et al.. The extensive variety of properties that were assessed were associated with the safety of their use, their capacity to withstand the harsh conditions of the human GIT and colonize it, to inhibit the growth of pathogenic bacteria and to possess functional potential. The results obtained revealed that promising probiotic candidates can be retrieved from spontaneously fermented products.

Spontaneous fermentations are driven by microbial consortia that are subjected to qualitative and quantitative changes, due to the dynamic nature of their microenvironment. Indeed, a succession at species and subspecies level is very frequently reported. However, studies assigning specific attributes of the final product to specific members of these microbial consortia, are generally lacking. In order to address this literature gap, Martini et al. investigated the bacterial dynamics at species and strain level during ripening of Parmigiano Reggiano cheese, which was fermented by natural whey starter, and the possible correlation with the evolution of the peptide profiles. The most frequently isolated non-starter lactic acid bacteria species were *Lacticaseibacillus rhamnosus, La. paracasei*, and *La. zeae*. More than 520 peptides were detected in the cheese samples, most of them originating from β-caseins. Occurrence of *La. zeae* was positively correlated with the incidence of 8 anti-hypertensive peptides. Similarly, Dong et al. studied the dynamics of the members of the microbial community during maturation of sauce-flavor Daqu of three colors, namely black, yellow and white and their effect on the compounds that affect the quality of the final product. It was reported that Daqu microecosystem consisted of fungi and bacteria, with the phyla Ascomycota and Firmicutes, respectively, being the most prevalent. Regarding fungi, genus *Thermoascus* was prevalent in all Daqu types. On the contrary, the bacterial diversity was more pronounced, with genera *Kroppenstedia, Virgibacillus*, and *Bacillus* being prevalent in black, yellow and white Daqu, respectively. Acidity was reported as the most important factor affecting the composition of the microcommunities. In addition, the key role of *Kroppenstedia* in color formation and of molds in pyrazine compounds formation were highlighted.

The development of autochthonous starter cultures is an emerging trend, which aims to improve the organoleptic quality of the final products and enhance their typicity and locality. Therefore, there is a need for studies assessing the potential of autochthonous strains and their effect on the quality of the final product. The studies by Grizon et al., Tzamourani et al., and Kamarinou et al. may serve as examples of studies that are aligned to this need. In the first study, the genetic and functional diversity of *Streptococcus thermophilus* strains isolated from different farms in the Saint-Nectaire cheese-producing PDO area in France, was investigated. A total of 22 non-commercial *S. thermophilus* strains were included in the study along with 4 commercial ones, and their genetic and technological properties were comparatively assessed. Pan-genome analysis revealed that the 41% of the genes could be characterized as hard-core genes, as they were present in 25 out of the 26 genomes, while the 56% of the genes could be characterized as accessory ones, as they were present in < 24 of the 26 genomes. This remarkable genetic diversity was partially exposed through the different acidification and proteolytic capacity of the strains. In the study by Tzamourani et al. a novel approach for rapid and efficient screening and classification of autochthonous yeast isolates with enological potential, was proposed. This approach allowed an effective technological classification by employing a phenotype-based, technologically oriented preselection procedure followed by biostatistical data treatment. The capacity of the proposed approach was validated and verified by micro-fermentation trials. Finally, in the study by Kamarinou et al., Feta cheese was produced by employing a commercial lactococcal starter culture that was supplemented with a mixture of autochthonous lactic acid bacteria strains belonging to the species *Lactococcus lactis, Levilactobacillus brevis, La. paracasei, Lactiplantibacillus plantarum*, and *Leuconostoc mesenteroides*. The final product was characterized by enhanced quality and safety, and distinctive organoleptic characteristics.

In conclusion, although the microbial communities of fermented products have been extensively assessed, especially in the case of products with commercial significance, novel approaches and insights are frequently presented. These, apart from improving our understanding of microecosystem development, reveal new possibilities and alternatives for future research.

## Author contributions

PP: Writing –original draft, Writing –review & editing. SP: Writing –original draft, Writing –review & editing. MA: Writing –original draft, Writing –review & editing. TB: Writing –original draft, Writing –review & editing. DA: Writing – original draft, Writing –review & editing. DT: Writing –original draft, Writing –review & editing.

